# Exploring the Use of Selection, Optimization, and Compensation Strategies Beyond the Individual Level in a Workplace Context – A Qualitative Case Study

**DOI:** 10.3389/fpsyg.2022.832241

**Published:** 2022-02-10

**Authors:** Iben Louise Karlsen, Vilhelm Borg, Annette Meng

**Affiliations:** National Research Centre for the Working Environment, Copenhagen, Denmark

**Keywords:** SOC strategies, IGLO, senior workers, successful aging strategies, developmental psychology, sustainable workplaces, organizational psychology

## Abstract

Due to aging populations and the prolonging of working lives, the number of senior workers will increase. Therefore, this study investigates the use of SOC strategies (Selection, Optimization, and Compensation) across organizational levels as a means for senior workers to maintain workability and age successfully at work. The need to expand the perspective of the SOC model beyond the individual level, when applied to a work context, has been emphasized theoretically in the literature, nevertheless, SOC strategies have so far only been examined at the individual level. This study is the first to explore SOC strategies at the organizational, leadership, and group level. We focus on senior employees and the SOC strategies they use to balance out demands and limited resources. Based on 23 semi-structured interviews with senior employees and immediate managers at two hospitals (nurses), and two dairies (skilled/unskilled workers), we explore which specific SOC strategies are used at each level and reflect on the applicability of broadening the perspective of the SOC model when applying it to a work context. Based on the empirical findings and the discussion of the empirical exploration of SOC strategies beyond the individual level, we argue that it is advantageous to further pursue this line of inquiry and include the group, leadership, and organizational level when applying the SOC model in a work setting.

## Introduction

Due to aging populations and the prolonging of working lives (Bal et al., 2015), the number of senior workers will increase. Prolonging work-life can, nevertheless, pose a challenge in some industries as some senior employees are challenged by reduced functional ability due to wear and tear, age-related physical and cognitive changes, and/or health problems (Ilmarinen, 2009). Studies have indicated that “successful aging” strategies in terms of selection, optimization, and compensation (SOC) are associated with occupational wellbeing and the maintenance of workability, by creating a balance between work demands and resources at work (see Moghimi et al., 2017 for a systematic review and meta-analysis). In this article, we want to take the first steps exploring how SOC strategies are manifested in the everyday working life at all four organizational levels and reflect on the relevance of broadening the perspective of the SOC model when applying it to a work setting. This exploration may serve as a point of departure for how companies and researchers may examine and work with SOC strategies across organizational levels.

The SOC model (Baltes and Baltes, 1990) comprises three types of strategies: Selection, referring to the individual setting of goals, and prioritization of goals, as a response to a reduction in resources, such as reduction of physical and/or cognitive functioning. The SOC literature (Baltes and Baltes, 1990) distinguishes between two types of Selection: “loss-based selection,” which refers to the involuntary abandonment of goals or tasks, and “elective selection,” which refers to a voluntary selection or prioritization of tasks or goals based on personal motives and preferences. Optimization, referring to the investment of resources (e.g., training, acquiring knowledge, or competencies), and effort to reach the goal or solve the task, and finally, Compensation, referring to the use of alternative means or external resources to reach the goal or solve the task. Selection, optimization, and compensation are strategies to advance the maximization of gains and minimization of losses associated with aging, and as a result promotes successful development and aging (Baltes and Baltes, 1990). As a theoretical model, however, the SOC framework does not limit itself to dealing with successful aging, it is a life-span model of successful development (Baltes and Carstensen, 1996; Baltes, 1997; Freund and Baltes, 2000) and can, in principle, be applied to a variety of domains and phases of the life cycle (Baltes and Dickson, 2001). Wiese et al. (2000) were some of the first to examine the usefulness of SOC strategies in the context of work and over the past two decades, organizational scholars have emphasized the potential benefits of applying the SOC model in a work context. Accumulated research has indicated that the use of SOC strategies has positive implications for the individual employee as well as the organization (Moghimi et al., 2017). Employees’ use of SOC strategies has been associated with a number of positive outcomes such as maintenance of professional competencies (Abraham and Hansson, 1995), with employees’ belief in future opportunities in the job (Zacher and Frese, 2011), job satisfaction (Yeung and Fung, 2009; Schmitt et al., 2012), with improved workability (Müller et al., 2012; von Bonsdorff et al., 2014; Weber et al., 2018; Žmauc et al., 2019), and wellbeing (Wiese et al., 2002; Carpentieri et al., 2017).

Transferring the model to a workplace setting focusing on senior employees, the SOC model has additionally proved useful in understanding how senior employees handle their everyday job tasks despite reduced functional ability (e.g., Müller et al., 2012). So far, the individual use of SOC strategies has proven beneficial in a work context (Baltes and Carstensen, 1999; Baltes and Dickson, 2001; Moghimi et al., 2017). However, in a growing number of papers, researchers have argued for the need to broaden the perspective of the SOC model beyond the individual level when applying it to a work context (Müller et al., 2016; Rudolph, 2016; Moghimi et al., 2017, 2019). The main argument for broadening the perspective is that the context and possibilities for applying the strategies change when the SOC theory is transferred from the private sphere to a work context. In the private sphere, the prioritization and abandonment of tasks (selection), the investment of more resources such as time and effort to complete the task (optimization), and the activation of alternative means to complete the task, for example, the acquisition of physical aids (compensation), are primarily up to the individual. However, in a work context, the freedom to prioritize, reorganize tasks, and so forth are more limited and often influenced by others. In a work context, all tasks have to be carried out, which means that the individual’s use of selection is limited and depends on the immediate manager’s acceptance or the willingness of one’s co-workers to take over the task. In a work context, there are deadlines or limited time assigned to complete tasks, minimizing the opportunity to, for example, spend extra time solving a task (optimization). Finally, if an employee, for example, requires a physical aid to complete a task (compensation), it would often have to be approved, ordered, and distributed by someone responsible for this in the organization. However, exploring SOC strategies at all organizational levels may not only reveal the more limited possibilities for applying the SOC strategies, it may also expose potentials for SOC use beyond the individual level to balance demands and resources within an organization.

Despite the theoretical argumentation for exploring SOC strategies across organizational levels (e.g., Moghimi et al., 2017), only a few studies have attempted to include other organizational levels than the individual when working with the SOC model. One of them is von Bonsdorff and colleagues, who use the employees’ average use of SOC strategies, based on each employee’s individual use, in their analyses (von Bonsdorff et al., 2018). In addition, only a few studies have explored the manifestations of SOC strategies at work, and these have only included the individual level (Müller et al., 2012, 2013; Ng and Law, 2014). Thus, knowledge is still lacking on the manifestation of the collective use of SOC strategies in workgroups and which SOC strategies are applied at the leadership level – both as direct SOC strategies from the manager and as actions that can support the use of SOC at the individual or group level. In-depth knowledge of which SOC strategies are used at workplaces at the various organizational levels is an important starting point for understanding what employees do to balance out demands and resources, potentially enabling them to maintain workability, despite functional decline or lack of resources. Further, knowledge about this field may serve as inspiration and a point of departure for future workplace interventions aiming to maintain workability by focusing on how SOC strategies at various levels of an organization may help balance demands and resources for employees.

To gain insight into how SOC strategies are manifested at the various organizational levels, the present study explores the SOC strategies used at the individual level as well as the organizational, leadership, and group level focusing on senior employees and the SOC strategies they use to balance out demands and limited resources. The aim of this study is, therefore:

(1) To get insight into if and how SOC strategies are manifested in the everyday working life at all four organizational levels.
(2) To reflect on the relevance of broadening the perspective of the SOC model to include the group, leadership, and organizational levels when applying it to a work context, and to discuss the empirical support for this broadening of the perspective.

## Materials and Methods

### Study Design and Population

Aiming to explore SOC strategies at all four organizational levels, we used a qualitative case study design. We chose two different kinds of workplace settings; hospitals and dairies.

Hospitals (nurses) were included because of the general risk of early retirement (Midtsundstad, 2004; Nilsson et al., 2011). Furthermore, nurses have high physical, cognitive, and emotional job demands and may be expected to use SOC strategies related to all three types of job demands. The two hospitals included in the study were large public hospitals (approx. 4,000–9,000 employees). To further explore the SOC strategies in relation to physical job demands, we included two dairies in the study. Dairy work is largely characterized by hard physical work, and there is a lot of repetitive work. Numerous studies have shown that early retirement is linked to low socio-economic jobs with high work demands (particularly physically demanding work), high workload, tight schedules, low control, and poor opportunities for development at work, etc (Elovainio et al., 2005; Lund and Villadsen, 2005; Thorsen et al., 2012; Poulsen, 2015). Unskilled dairy work has many or all of these characteristics, and the workers are at increased risk of early retirement. Dairies do, however, include both unskilled and skilled workers such as technicians and dairymen. The two dairies were one dairy with about 130 employees being part of a larger company and one private company with about 60 employees. In addition, the four workplaces were selected to obtain a variety in size, organizational structure, and geographical location.

### Recruitment of Participants

Across all four workplaces, we recruited senior employees and immediate managers with personnel responsibility of senior employees. Senior employees were defined as above age 55 for two reasons; firstly, at the dairies, the labor union agreement required the workplaces to have a senior policy that included employees at age 55 and above, focusing on keeping these employees at the workplace. Consequently, “senior employees” were already a preexisting category at these workplaces defined as being above age 55. Secondly, we worried that raising the age criteria for inclusion might have produced problems with a “healthy worker” selection so that the senior employees who needed to use the SOC strategies might already have left the job due to reduced functional ability. Therefore, we chose to recruit employees from the relatively young age of 55 years.

Different recruitment strategies had to be applied due to structural differences in the two types of workplaces and different access to key stakeholders. At the dairies, the managers recruited the participants, which were production workers or support functions to the production line (e.g., machine maintenance). We had provided the managers with a description of the research project. We asked to talk to senior employees about topics related to balancing demands and resources at work. As the included dairies were small and medium-sized enterprises with few senior employees, we interviewed practically everyone over 55 at these workplaces.

At the first hospital, a coordinator recruited nurses from different hospital units. This approach was, however, not possible at the second hospital. Instead, an announcement was placed on the intranet encouraging nurses to contact us if they were interested in participating in the study. The last approach resulted in very dedicated interviewees who chose to spend their free time contributing to the study; however, the data from their interviews did not differ substantially from the data from the other interviewees. All interviews were treated confidentially, ensuring that individuals could not be identified in the results, and data were handled according to the Danish Data Protection Authority guidelines. A total of 47 participants, 30 women and 17 men, were included in the study. The uneven gender distribution in the study was due to the overrepresentation of women among the nurses. Of the 47 participants, 26 were employed at the dairies, and 21 were employed at the hospitals. We interviewed a manager at each of the four workplaces; the remaining 43 interviews were with employees. The participants were aged between 55 and 73 years. They all had permanent positions.

### Interviews

To gain the benefits of group discussions and the more anonymous setting for in-depth discussions of more sensitive matters, we applied a mix of focus groups and individual interviews.

We aimed to complete a focus group interview at each workplace with 6–8 employees of approx. 1.5 h duration, and five semi-structured individual interviews of approximately 1 h each - four with employees and one with a manager responsible for senior employees. At one workplace, one of the interviewees did not show up for the individual interview. Consequently, the study is based on 19 individual, semi-structured interviews and four focus group interviews. All interviews were conducted at the workplace in a quiet room. At the beginning of all interviews, the interviewees were informed about the purpose of the study, that the interview was recorded, and that they did not have to answer questions that they did not feel like answering, and thereby provided informed consent to participate in the study. The first and the last author, an anthropologist and a psychologist, carried out all interviews.

### Data Collection Procedure

To explore the use of SOC strategies at all four organizational levels, a semi-structured interview guide was developed. The participants were encouraged to describe their everyday work challenges, how they went about their work tasks and overcame these challenges, and what was done at each organizational level. In addition, the participants were invited to reflect on what factors affected the possibilities for using the different types of strategies at work that they mentioned and encouraged them to make their descriptions as specific as possible. To explore the use of SOC strategies more explicitly, we asked questions addressing the use of SOC strategies we knew from the literature and SOC strategies we had heard about in earlier interviews. As a consequence, the interview guide combined more open questions, such as; “which challenges do you experience as a senior worker at this workplace – and what do you do to overcome those challenges?” with inquirers into the use of specific SOC strategies such as “do you have the option to get/use technical assistive devices at work?” or “is it possible to apply more time solving particular tasks?” (see interview guides in Supplementary Appendix 2). At each workplace, we began the data collection process with the focus group interview. The interview guide was adjusted before returning to the workplace for the individual interviews. Adjustments were made according to the more detailed knowledge of the employee’s job and issues that we wanted to explore further. In this way, the data collecting was a dynamic process that developed as our knowledge of the workplaces grew (Wadel, 1991).

### Data Analyses

As our main analytic framework, we used the SOC model (Baltes and Baltes, 1990). We operationalized the three types of strategies based on the existing literature, particularly Müller et al. (2012) who had conducted a qualitative exploration identifying job-specific manifestations of SOC in nursing, was useful when operationalizing the strategies in a workplace setting (Müller et al., 2012, p. 1635). The three types of strategies were operationalized in a process in which we moved back and forth between the theoretical description of selection, optimization, and compensation and the empirical material and adjusted the descriptions of the codes. We continued adapting the code descriptions until we had three definitions that allowed us to code the entire data material individually and compare the coding.

In addition to the operationalization of the three types of SOC strategies, we used the IGLO framework (Day and Nielsen, 2017), which identifies an organization as consisting of four levels – individual, group, leadership, and the organizational level, to classify the strategies according to the organizational levels. As we were interested in exploring whether SOC Strategies could be found across all organizational levels and how they manifested themselves, the analytical approach was deductive, using a predefined coding tree consisting of selection, optimization, and compensation (as defined in Table 1) at each organizational level.

**TABLE 1:** Operationalization of selection, optimization, and compensation – definition used to code the empirical data material.

Type of strategy	Operationalization of the strategies
Selection	The selection of work tasks and areas of responsibility, new positions in the company. Delegation of work tasks to colleagues. Do one thing at a time
Optimization	Activities to optimize one’s resources to perform the task (possibly the selected tasks). Courses, training, awareness of ergonomics, waiting until you are completely healthy to return from sick leave, plan work efficiently, read literature, and stay up to date with knowledge relevant to the task
Compensation	The use of aids or other alternative means to be able to solve the task. For example, compression stockings, lifts, raising/lowering tables, trolleys, help from colleagues, use a chair during standing work, adjust the execution of the work task to make it less straining, and spend more time on the work tasks

The interviews were all recorded, transcribed, and coded in Nvivo 11. The first and the last author coded the empirical data individually and compared the codes. Inconsistencies were primarily found concerning the categorization of optimization and compensation strategies, as it can be difficult to distinguish between the two types of strategies in practices. The way you, e.g., lift, can be both compensation and optimization, depending on why you adapt your behavior. If an employee adapts a behavior due to pain, we considered it compensation, but the behavior could also reflect general considerations of work ergonomics. In this case, we coded the behavior as optimization. We considered the context and the circumstance described in the interview when coding the SOC strategies. In cases of ambiguity about the correct categorization of a strategy, the first and the last author met to discuss the coding and examined the context and the intention of using the strategy as explained in the interview by the employee or manager.

It caused little difficulty identifying which organizational level had initiated using a given strategy examining the individual and the group level. However, it was not always clear to some of the employees if a strategy were initiated at the leadership or the organizational level. Most often, employees would learn about the strategy from the immediate manager, not always knowing if the initiative originated from this level or the organizational level. We identified a strategy as belonging to a certain organizational level based on the information provided in the interviews.

## Results

Below, we present an overview of the number of SOC strategies we encountered in the interviews across the four organizational levels divided into the two industries (Table 2), and we provide examples of the specific manifestations of the SOC strategies used at each of the organizational levels. Subsequently, we discuss challenges associated with the use of the expanded perspective of the SOC model in general.

**TABLE 2:** Overview of the number of occurrences of SOC strategies at each of the organizational levels in the data.

	Selection (Hospital/Dairy)	Optimization (Hospital/Dairy)	Compensation (Hospital/Dairy)	Total SOC at each level (Hospital/Dairy)
Organizational level	11 (5/6)	32 (14/18)	6 (4/2)	**49 (23/26)**
Leadership level	25 (18/7)	3 (3/0)	0 (0/0)	**28 (21/7)**
Group level	10 (4/6)	18 (4/14)	1 (1/0)	**29 (9/20)**
Individual level	23 (14/9)	46 (17/29)	11 (5/6)	**80 (36/44)**
Total number of strategies across organizational levels	**69 (41/28)**	**99 (38/61)**	**18 (10/8)**	**186 (89/97)**

*Line and colon totals are highlighted in bold.*

### The Manifestation of Selection, Optimization, and Compensation Strategies at the Four Organizational Levels

Selection, optimization, and compensation strategies were encountered at all of the organizational levels, except compensation at the leadership level, illustrating that SOC strategies are applied beyond the individual level. Nevertheless, we encountered most examples of SOC strategies at the individual level followed by SOC strategies at the organizational level, while we encountered fewest examples of SOC at the leadership and group levels. Comparing the three types of SOC strategies, optimization strategies were the most common in the data followed by selection, and only relatively few examples of compensations strategies (see Table 2). Table 2 presents the number of occurrences of a given SOC strategy at a given level. Only SOC strategies used at work or in connection to the workplace (e.g., running with co-workers after work) were included in the data. For an overview of selected examples of specific strategies found at each level, see Supplementary Appendix 1.

Table 2 further shows different trends in the two industries. In terms of organizational levels, SOC strategies at the leadership level occurred more than twice as often in data from the hospitals than dairies. On the contrary, SOC strategies at the group level occurred twice as often in the data from the dairies than the hospitals. When we look at the type of strategies used, we find that selection occurred more in data from hospitals than in dairies (especially at the leadership and individual level). In contrast, optimization strategies occurred a lot more in data from dairies than hospitals.

In the following, based on our data, we define the use of SOC strategies at each organizational level. Based on our empirical data, we will give concrete examples of selection, optimization, and compensations at each organizational level, thereby providing a general definition and specific, contextual insight into how SOC strategies may be manifested at each organizational level.

### Selection, Optimization, and Compensation Strategies at the Organizational Level

In the study, we operationalized the organizational level as including top management, Human Resource Management (HRM), corporate bodies such as Environment-Health and Safety (EHS) representatives, and the Working Environment Management Organization. The empirical examples indicate that the use of SOC strategies at this levels should be defined as a span from strategies used directly by these actors to these actors merely creating or securing a context facilitating the use of SOC strategies at other levels. An overview of the SOC strategies at the organizational level is shown in Supplementary Appendix 1.

At this level, selection strategies appeared to mainly manifest themselves as the organization found new positions or created new positions to keep employees that were no longer able to maintain their old positions. A concrete example was an employee who, according to her wishes, was moved from a department with evening and night shifts to one with only day shifts to avoid the extra burden of the changing work hours.

Optimization at the organizational level manifested itself as the organizations buying technical assistive devices to ease the burden on the employees as well as arranging activities that supported the health and wellbeing of the employees such as access to massage and health-promoting activities such as a bike trip. Optimization at this level was also manifested as the organization ensuring that employees could attend training or education programs to maintain competencies or making sure that employees worked as ergonomically correct as possible.

The most common manifestation of compensation at the organizational level was to provide a technical assistive device as a direct response to a particular need among one (or more) of the employees. A concrete case was that top management decided to invest in adjustable chairs in some departments because some employees suffered from pain in the upper back, neck, and shoulders. Furthermore, compensation strategies at this level were to make physiotherapy, psychological help, or other health insurance available to the employees.

### Leadership Level

At the leadership level, we refer to the immediate manager. The empirical examples indicate that the use of SOC strategies at this levels should be defined as a span from the immediate manager’s active partaking in executing the strategies to merely supporting the group or the individual in using various strategies. An overview of the SOC strategies at the leadership level is shown in Supplementary Appendix 1.

Selection at this level manifested itself as the immediate manager relieved employees from stressful tasks or exchanging their tasks if they experienced a decline in their functional abilities. A specific example was an immediate manager who was aware that an employee could not accommodate as much as previously and therefore assigned her fewer patients than before.

Optimization at the leadership level manifested itself as the immediate manager striving to fulfill the employees’ requests when making the employees’ work schedules to optimize their recovery. This could for example, be a nurse requesting to have an extra day off following a nightshift. Optimization at this level could also be that the immediate manager was aware of employees’ competencies and challenges and organized the work making sure that the competencies were used in the best possible way and that strain on the employees was minimized.

We did not encounter any cases of compensation at the leadership level.

### Group Level

At the group level, we refer to one’s closest group of co-workers. Defining one’s group might be difficult due to job rotation, working in shifts, and so forth. In the interview guide, the questions were phrased to make the participants think of the co-workers he/she “would usually work with.” The empirical examples of SOC strategies indicate that the use of SOC strategies at this levels should be defined as a span from the collective use of shared strategies in the group to merely supporting each other in using the strategies at the individual level. An overview of the SOC strategies at the group level is provided in Supplementary Appendix 1.

Selection at the group level mainly manifested itself as the co-workers redistributing job task within the group to relieve co-workers who needed it; it could be the group agreeing that a co-worker with back pain was exempted from a burdensome workstation in the rotation system to relieve him/her of the strain.

Optimization at the group level manifested itself as arranging job rotation to create variation in the work tasks and to remind each other not to strain the body unnecessarily doing work as well as doing exercise together during a break or getting together after work to go cycling. Such exercising activities might, of course, not purely serve the purpose of staying fit to maintain workability, but are perhaps done for social reasons, but had optimization as a side win. Other optimization strategies at the group level were to share knowledge and support each other professionally and help each other to stay updated on a given field of expertise.

Compensation at the group level manifested itself as the group members helping each other with work tasks, and in this way, compensate for each other’s reduced functional capacity. This was used widely across all four workplaces.

### Individual Level

At this level, as in the original SOC model, it is the individual who initiates and uses the SOC strategies. As the individual level is rather well-explored in the SOC literature, we merely supplement existing knowledge at this level with additional insights, from our empirical data, on the specific manifestations of SOC strategies used by the individual. An overview of the SOC strategies at the individual level is shown in Supplementary Appendix 1.

At the individual level, we encountered selection strategies already described in the existing SOC literature: employees prioritizing among or delegating work task, deselected leisure activities, or doing one task at a time (Abraham and Hansson, 1995; Müller et al., 2013; Ng and Law, 2014; Zacher et al., 2015). We did, however, also encounter new selection strategies, which have not been presented in existing SOC literature: employees applying for positions in other departments of the organization (e.g., a nurse applying for a job at the outpatient clinic to avoid night shift), employees who took on new/other responsibilities in the organization, such as work environment representative to get more administrative work and reduce time spent on production work in the dairy, and finally, employees declining to take on extra work or responsibilities.

In regards to optimization, we encountered many strategies that are already described in the existing SOC literature (Ibid.), such as doing exercise, staying updated in one’s field of expertise, and using the body ergonomically correct. Furthermore, we encountered many examples of employees using technical assistive devices, not as compensation strategies to compensate for functional decline, but to prevent wear and tear, and thus optimizing their available resources. Following the rational that using one’s body ergonomically correct is regarded as an optimization strategy (Müller et al., 2012), we likewise categorized the use of technical assistive devices when used for preventive purposes as optimization. Subsequent, the use of technical assistive devices can be either optimization or compensation depending on the reason for the use of the strategy. Another example of optimization not previously described in the literature ensure recovery, such as asking for an extra day off following a nightshift by.

Concerning compensation, we only encountered strategies that are already described in the SOC literature (ibid.): using various technical assistive devices, using your body slightly different while working, and getting some kind of treatment, such as physiotherapy. See Supplementary Appendix 1 for a list of specific examples of strategies used at this level.

## Discussion

In this article, the purpose was to take the first step toward investigating the relevance of working with an extended SOC model when studying workplaces. Expanding the SOC model to other levels than the individual may initially seem incompatible with the original SOC model’s idea, which aims to understand how the individual can balance demands and resources throughout the lifespan. However, when focusing on a workplace context, as we have done, it appears necessary to broaden the focus and examine the impact of the group, leadership and organization. Not at the expense of the individual but to understand how the individual, in this context, is given the opportunity (or denied the opportunity) to balance demands and resources, as proposed theoretically by several researchers (Müller et al., 2016; Rudolph, 2016; Moghimi et al., 2017, 2019). Examining the strategies used at four workplaces, we found that SOC strategies are used at all four organizational levels, only we did not find examples of compensation strategies at the leadership level. In this discussion, we will reflect further on the relevance of broadening the perspective of the SOC model in a work context and discuss the empirical support for broadening the model.

### What Does It Add to Explore the Use of Selection, Optimization, and Compensation Strategies Beyond the Individual Level?

Exploring SOC strategies beyond the individual level expands the focus on types of strategies that are used and can be applied to ensure that all organizational levels support senior employees’ workability and successful aging at work – supporting a healthy workplace for all employees across age groups. It has been argued elsewhere (Day and Nielsen, 2017, p. 302) that to have a healthy organization, there should be a balance of initiatives that look at the overall organization, groups, leaders, and individual employees. An over-reliance on or a single focus on only one of the levels (e.g., a focus solely on the individual) may create an imbalance and the perception that the problem is always at the individual level without trying to address the issue at a higher organizational level (Ibid). When exploring SOC strategies across the four organizational levels, it is essential to stress the interconnectivity between the levels as well as the internal hierarchy of the levels. For instance, we encountered the individual selection strategy “to choose not to take on any extra work” to avoid overburdening oneself. However, exploring the group level revealed that feelings of guilt or indirect social pressure could hinder the use of this strategy in an everyday work context. Including the additional organizational levels when exploring the use of SOC strategies, reveals how the individual constantly has to negotiate strategies with others (directly or indirectly) before applying them or are dependent on others to make particular strategies available to use. Elsewhere in the data, the interplay between levels showed that there is not always need for intervention at the leadership level because a given problem with high workload in the production was already solved internally at the group level through job rotation between employees (i.e., a SOC strategy at the group level). Thus, it may not always be a requirement that strategies are applied at all the organizational levels when addressing a potential source of job strain. For these reasons, we recommend that the organizational levels should be explored and viewed as interconnected in future studies.

### Differentiating Between Selection, Optimization, and Compensation Strategies Beyond the Individual Level and Job Design/Job Crafting

Job design (Hackman and Oldham, 1976) refers to the characteristics of the job, which are more or less static boundaries. It is a top-down process where management designs the job and thus decides on its characteristics. Job crafting complements the job design perspective by showing how employees can actively shape their work conditions within these given boundaries. Job crafting is defined as the physical and cognitive changes individuals and teams (“team job crafting”) make to the task or relational boundaries of their work (Wrzesniewski and Dutton, 2001; Tims et al., 2013). Job crafting can take three different forms: (1) Task crafting, which refers to altering the form and number of activities one engages in while doing the job. (2) Relational crafting which refers to exercising of discretion over with whom one interacts while doing the job. (3) Cognitive crafting which refers to altering the way one sees the job. Wrzesniewski and Dutton (2001) list the following three needs or motivations for employees to engage in job crafting: Firstly, to assert control over their jobs to avoid alienation from the work, secondly, to create a positive self-image in their work, and third, to fulfil the human need for connectedness to others. Thus, job crafting is strongly connected to the employees’ identity and identification with their job. The SOC model may be regarded as a form of job crating. Like the job crafting perspective, the SOC model also regards the employees as active co-creators of their work conditions. However, job crafting focuses on creating a positive job identity and meaning in the job, whereas SOC strategies are ways to respond to limited resources. When looking at the expanded version of the SOC model, SOC strategies at the organizational and, to some extent, the leadership level may be closer to the job design perspective where SOC at the individual and group level may be regarded as closer to job crafting.

### Exploring Selection, Optimization, and Compensation Strategies Across the Work-Life

The original SOC model is a lifespan model where the SOC strategies are responses to reduced resources to minimize losses and maximize gains throughout the lifespan (Baltes and Baltes, 1990). In our study, we only focused on senior employees to explore the strategies used to maintain workability. However, the interviews revealed that SOC strategies, at all organizational levels, could be relevant across all age groups as one could apply the SOC strategies throughout one’s work-life as resources are affected by both internal (e.g., pain) and external (e.g., time pressure) circumstances. A nurse explains that the use of SOC strategies is not just relevant in relation to older nurses: “We had a colleague who had had a shoulder operation. She wanted to start work shortly after the operation. Therefore, we had to show consideration and make it work, despite the reduced work capacity.” Other SOC researchers (Müller et al., 2012) have likewise stressed that optimization in the form of lifting correctly can prevent wear and tear among nurses, regardless of age, and foster wellbeing and workability of nurses. Furthermore, the relevance of using SOC strategies to improve the work-life balance has been stressed (Baltes and Heydens-Gahir, 2003; Unger et al., 2015). This might be of particular relevance, for example, when starting a family or when a pandemic suddenly causes many to work from home, creating a need to restore the balance between work and family life. This further supports the relevance of using SOC strategies throughout the entire work-life. Therefore, we propose exploring the SOC strategies beyond the individual from a lifespan perspective at work and encourage researchers to examine the use of the strategies throughout different work-life stages. Focusing on SOC strategies for senior employees to stay employed longer, it is, however, likewise important, not only to look at what can be done in the latter part of one’s career but throughout the entire work life to prevent wear and tear and other work-related challenges among senior employees. Our study thus supports that the SOC model is best regarded as a life-span model of successful development (Baltes and Carstensen, 1996; Baltes, 1997; Freund and Baltes, 2000).

### Selection, Optimization, and Compensation as a Response to Organizational Changes as Well as Developmental Changes

During the interviews, we often encountered the issue of changing working conditions. When we asked participants to recall changes regarding their resources at work, they often found it hard to distinguish between which changes they had themselves undergone and which changes their profession had been subject to. Since the participants first entered the labor market, it was perceived to have changed dramatically; at the dairies primarily in regards to the growing use of new technology, automation, and robots. In nursing changes were primarily experienced in regards to professional development, development in equipment, medicine, IT, types of patients/illnesses, work pace, and hospital occupancy. All such changes might, along with the life stage changes, require temporary use of SOC strategies. Thus, the use of SOC strategies, at the four organizational levels, may not only help employees deal with reduced resources caused by developmental changes but also to deal with external changes, such as changes to one’s profession. The expanded SOC model with several organizational levels seems particularly suitable for a work context, as modern workplaces are undergoing constant changes, where there is a need to handle both individual developmental changes and organizational changes, which is best done by involving several organizational levels in a workplace.

### Strengths and Limitations

Methodologically, aiming to get in-depth knowledge of the SOC strategies used at the different organizational levels meant that we focused on fewer cases and only included four workplaces in the study. Therefore, more cases are needed to support the findings of this paper further. In future studies, we would recommend including a wider age group to explore how SOC strategies, at the four organizational levels, manifest themselves at various life-stages of the employees.

Even though we recruited nurses whose work is characterized by physical, cognitive, and emotional job demands, we primarily encountered SOC strategies connected to physical demands. The reason why we only found a few cases of SOC strategies used to overcome cognitive challenges was most likely due to our participants being relatively young, and therefore not likely to experience notable problems with their cognitive functioning. Furthermore, it is likely that employees in these job categories (nurses and skilled/unskilled workers) would leave their jobs due to physical challenges before reaching a critical stage of cognitive challenges. To provide more insight into the manifestation of SOC strategies related to cognitive demands, it might be necessary to include white-collar workers, perhaps at administrative or academic workplaces. One might turn to younger employees, to study the manifestation of SOC strategies used to overcome emotional job demands. This kind of demand is likely to be associated with the nursing discipline, and there might have been an adaptation to this as a senior employee. Alternatively, those experiencing high emotional job demands might have chosen a different career path early, which might explain why we did not see any cases of this among the senior employees. Nevertheless, despite these limitations, this is the first study exploring the manifestation of SOC strategies at multiple organizational levels empirically, and thus provide new knowledge on this field of research.

## Conclusion

We sat out to explore SOC strategies beyond the individual level focusing on how senior employees balance out demands and limited resources, supporting a longer work life. We encountered specific manifestations of SOC strategies across all organizational levels, only did we not find any examples of compensation strategies at the leadership level. These findings imply that there is empirical support for pursuing this line of enquiry further.

Looking beyond the individual level may help overcome limitations posed to the individual and reveal unique opportunities for the collective use of SOC. In this article, we took the first steps exploring how SOC strategies are manifested in the everyday working life at the individual, group, leadership, and organizational level. Furthermore, we empirically tested the relevance of broadening the SOC model to include these other organizational levels in a work context. Thus, the study contributes with a point of departure for how companies and researchers may examine and work with SOC strategies across organizational levels to create a balance between demands and resources throughout the working life, potentially supporting a longer and healthier working life. We suggest that exploring SOC strategies at all organizational levels can contribute to a more social and context-oriented approach to future research in the field.

## Data Availability Statement

The datasets presented in this article are not readily available because due to the Danish Data Protection Act, data cannot be made public available. Researchers interested in access to the data are encouraged to contact the corresponding author. Requests to access the datasets should be directed to IK, ika@nfa.dk.

## Ethics Statement

Ethical review and approval was not required for the study on human participants in accordance with the Local Legislation and Institutional Requirements. Written informed consent for participation was not required for this study in accordance with the National Legislation and the Institutional Requirements.

## Author Contributions

IK and AM conducted the interviews, coded and analyzed the empirical data, and revised and developed the manuscript. IK wrote the first draft of the manuscript. AM designed the study and obtained the funding in collaboration with VB and two former colleagues (see section “Acknowledgments”). VB provided professional input throughout the project and was invited to comment on the manuscript during the writing process. All authors contributed to the article and approved the submitted version.

## Conflict of Interest

The authors declare that the research was conducted in the absence of any commercial or financial relationships that could be construed as a potential conflict of interest.

## Publisher’s Note

All claims expressed in this article are solely those of the authors and do not necessarily represent those of their affiliated organizations, or those of the publisher, the editors and the reviewers. Any product that may be evaluated in this article, or claim that may be made by its manufacturer, is not guaranteed or endorsed by the publisher.
