# Supplementary material for: Exploring the Use of Selection, Optimization, and Compensation Strategies Beyond the Individual Level in a Workplace Context – A Qualitative Case Study
Source: Front Psychol. 2022 Feb 10;13:832241. doi: 10.3389/fpsyg.2022.832241 (PMC8866242; doi:10.3389/fpsyg.2022.832241)
Supplement: Supplementary file 1 [file Data_Sheet_1.pdf]

Appendix A. List of SOC strategies used at the organisational, management, group, and individual level from the interviews

Type of SOC strategy	Overall strategy	Concrete examples from the interviews
Organisational level		
Selection	-Persons at the organisational level find or create new positions in the organisation for employees who can no longer manage their current position	-An employee who could no longer work nightshifts was offered a position in another department in the dairy where he was only required to work dayshifts.<br>-Management created an outpatient clinic specialising in wounds which a nurse was made responsible for, to relieve her of the physical strain of her previous position.
Optimisation	-Buy aids to reduce the strain on the employees	-Management at the dairy has bought an elevation platform so the employees did not have to lift above shoulder height.
	-Enforce the use of aids	-Employees are given a warning if they lift heavy items without using the required lifting aids.
	-Focus on ergonomics	-Management had an occupational therapist go through the dairy and work out recommendations for improvements of the work procedures.
	-Arrange massage, physiotherapy and/or health promoting initiatives	-A department at a hospital had arranged for a masseur to come every other week and give the employees a short massage.<br>-A dairy organised a health year where various activities such as walks, runs, bike trips, and work out was arranged.
	-Initiatives to maintain and develop the competencies of the employees	-A dairy had a pool of money devoted to covering the cost of courses for the employees.

**Compensation**	-Buy aids as a response to functional impairment of employees<br>-Provide access to treatments	-Many of the employees had problems with pain in shoulders, neck and back. As a response to this, the hospital invested in special chairs for the employees.<br>-A workplace had a contract with a health insurance company so when the employees had an injury, they had free access to massage, physiotherapy, and chiropractor.
	Leadership level	
**Selection**	-Immediate manager relieves employees from straining work tasks or adjust their task/area of responsibility	-The immediate manager relieved an employee from a specific workstation in the production of the dairy because she had pain in her collarbone.<br>-The immediate manager is aware of a nurse who cannot cope with as much as before and gives her fewer patients than previously and is standard.<br>-The immediate manager assigns a nurse to a teaching task to reduce her physical strain. In doing this, the immediate manager took into consideration that the nurse had a lot of experience in the field that needed to be taught, and that she liked teaching.
**Optimisation**	-Immediate manager provides support for the employees' optimisation strategies<br>-Immediate manager has focus on minimising the	-An immediate manager explains how she takes the requests of the employees into consideration when organising the long term work schedule. The employees often have certain requests to ensure the best possible recovery after e.g. night shifts.<br>-An immediate manager tells how she, when planning the daily work schedule, always puts two nurses on the heavy patients to minimise the burden on the nurses.

	strain on the employees when organising the work	
**Compensation**	-No examples in the interviews	
Group level		
**Selection**	-Rearrange work task among the team members so colleagues who needs it, can opt out of tasks that overburden them	-If a colleague has difficulties standing, the workgroup agrees that he/she gets the "floor position" where it is possible to sit down every now and then, that day.<br>-If a colleague has back pain, the work group agrees that he/she can opt out of the most burdensome work station in their rotation routine, that day.
**Optimisation**	-The work group plans a rotation routine between work stations to create variation and minimise strain	-The employees at a machine in the production of a dairy rotate between three different work stations. They rotate after each break.
	-Train/do exercise together	-The colleagues encourage each other to do exercises in the breaks.<br>-The colleagues arrange joint cycle or walking trips after work.
	-Share knowledge/experience to keep updated within the field	-The nurses share knowledge with each other. The young nurses introduce new theories and the older nurses share their experiences from their many years in the field. One nurse also shares knowledge with the physicians.
	-Shared focus on ergonomics	-An employee makes his colleagues aware of it if he sees them e.g. lifting in a way that may hurt their back.

Compensation	-Colleagues help each other in the group with tasks that the colleagues cannot manage alone	-The employees talk about how you get various injuries as you age, and that they help each other in the work group with the tasks they have difficulties doing because of these. One nurse specifies that some days she cannot give injection because of pain in her hand and then her colleagues will give the injections for her.
Individual level		
Selection		
	-Apply for other positions either inside or outside the organisation	-An employee suffering from back pains applied for a position as a team leader in the department to reduce the physical burden at work.<br>-A nurse told that she had applied for a position in a less "heavy" ward.
	-Decline extra work or responsibilities	-A nurse avoids taking on extra shifts because she does not have the energy for it.
	-Assume certain responsibilities to reduce the amount of straining work	-A dairy worker volunteers to be "work environment representative" and this way avoids working evening and night shifts, because these responsibilities are during the day.
	-Delegate tasks to colleagues	-An experienced nurse has a very good overview of which tasks that needs to be done. However, to avoid overburdening herself, she assigns some of the tasks to her colleagues, when they are not busy, rather than doing all herself.

	-Completing one task at a time	-A dairy worker sees the young workers run about trying to do everything all at once. He explains that with ageing you learn to do one thing at a time, and this way makes the work easier for yourself.
	-Prioritise the most important tasks and drop less important tasks	-A nurse tells how she based on experience and information about the patient quickly identifies "need to do" and "nice to do" tasks and then prioritise the important tasks – she cannot manage all tasks.
Optimisation	-Use aids to reduce the work strain	-A dairy worker uses the elevation platform to avoid lifting above shoulder height. A nurse tells how she uses a trolley instead of carrying the heavy things herself.
	-Pay attention to using the body ergonomically correct while working	-One dairy worker always carries things close to the body to reduce strain to the back. Another tells how she takes a step to the side instead of twisting her body when lifting things from beside her.
	-Do exercise/work out	-A nurse with an office position makes up for all the sitting by making sure to take the stairs at least once a day, and park her car a ten to fifteen minutes' walk from work, or when taking the bus, gets of a stop too early.<br>-A dairy worker with knee and back problems does back exercises, cycles, and plays badminton to keep strong.
	-Focus on recovery	-Employees make requests for the work schedule to ensure enough recovery. For example ask to get two days off in a row after a night shift to recover fully, or request to have their day of in the middle of the week so they can recover between the working days.

		-Another employee stretches her back by lying down on the sofa for a while when she comes home from work.
	-Go for massage/physiotherapy etc.	-A nurse does back exercises and goes to a chiropractor clinic to be able to keep working.
	-Keep up with the field	-A diabetes nurse finds information about new insulin pumps on the net, in manuals, and by contacting the manufacturers.<br>-To be better able to handle patients with substance abuse, another nurse took a course in conflict management<br>-A nurse took a wound care course to specialize
Compensation	-Use individual aids to manage the work tasks	-A worker at the dairy uses a kneepad when he kneels at work due to a knee injury.<br>-Another employee uses a roller mouse because of pain in the shoulder/arm.
	-Ask colleagues for help to complete work tasks	-A nurse with osteoarthritis in her hands uses pliers or asks a colleague for help, when she has to open a screw cap.
	-Adjust the way of carrying out the work task	-A nurse has problems with her shoulders. To avoid lifting her arm above shoulder height, she has to stand up when she takes things from the shelf above her desk. She also tries to use her left hand (instead of the right) every now and then.

		-A worker at the dairy suffers from pain in the joints of her thumbs. Therefore, on bad days, she lifts with the little finger side of her hand instead.
	-Receive treatments	- Finally, several employees go to physiotherapy to relieve work related pain.

Appendix B. Interview guides – 1) individual interviews, 2) interviews with managers 3) focus group inter

INTERVIEW GUIDE – INDIVIDUAL INTERVIEWS

PRESENTATION	- How old are you?<br>- How many years have you been in this workplace?<br>- What job function do you have? / What are your work tasks?
DESCRIPTION OF THE WORK	- Describe the work in broad outline, what does it consist of?<br>- Do you work alone / with others?<br>- How much influence do you have on your tasks and the organization of work in general?
WHAT CHALLENGES DO YOU EXPERIENCE IN GETTING OLDER IN YOUR WORKPLACE?	- What do you do then? (when you experience these challenges in your work)<br>- How do you handle these challenges in your daily work?<br>- Is there anything you could do? (Promoting/inhibiting?)<br>- Is there anything your colleagues, managers are doing to deal with this? (Promoting/inhibiting?)
FROM THE INDIVIDUAL TO THE GENERAL	- Is what you experience a "common" problem when you get older in this workplace?<br>- Do you know if the management is doing something to try to change this? (why/why not?)<br>- (Have you encountered other challenges in getting older in this workplace among your colleagues?)
SOK ELEMENTS (PROMOTING/ INHIBITING)	- Do you have the opportunity to get/use aids that can help you in your work?<br>- Is it possible to attend courses?<br>- Is it possible to spend more time on the work tasks?<br>- Is it possible to use a physiotherapist / training (can you train at work?)
ARE THERE TASKS THAT YOU PERFORMED IN THE PAST THAT YOU DO NOT PERFORM TODAY?	- If so, why/why not?<br>- (Ask why this changed, on whose initiative it happened, and how the new tasks work)
ARE THERE WORK TASKS THAT YOU HANDLE TODAY THAT YOU HAVE NOT HAD BEFORE?	- What is the background for this change in the work tasks?<br>- (Ask why this changed, on whose initiative it happened, and how the new tasks work)

DO YOU HAVE A SENIOR POLICY IN THIS WORKPLACE?	- Do you think they want employees to stay in this workplace for a long time?<br>- If so, do you know what this policy is about?<br>- Do you make use of the opportunities the senior policy provides?<br>- If so, which ones?<br>- If not, why not?<br>- How do you experience that your colleagues, your immediate manager, your workplace generally perceive senior employees?<br>- How is this expressed?
HAVE YOU THOUGHT ABOUT WHEN YOU WOULD LIKE TO RETIRE?	- What is your motivation for retiring/staying at work?<br>- What could make you stay longer in this workplace?<br>- What would it take you to both *be able* and *wanting to* stay in this job?
IS IT COMMON TO STAY IN THIS WORKPLACE FOR A LONG TIME?	- How old are people typically, when they retire from this job?<br>- What do you typically think is the reason people stop at this workplace?<br>- When employees stop at this job, do they typically retire or do they get a new job?
FINAL COMMENTS	- In conclusion, is there anything you think is relevant in relation to this project of becoming older at work, which we have not yet touched on?<br>- Is there anything else you would like to add before we end?

INTERVIEW GUIDE – MANAGERS

PRESENTATION	- Which department are you head of?<br>- How many employees are you responsible for?<br>- How many years have you been a manager in this workplace?<br>- What are your responsibilities as a manager?<br>- How are you positioned organizationally as a leader? (other organizational levels above / below you)<br>- Do you have an influence on the organization of the employees' work / tasks?
SENIOR EMPLOYEES AT THE WORKPLACE	- How many employees are there in total at this workplace / in your department?<br>- Are there many employees over the age of 55 in this workplace / in your department?<br>- What are the benefits of having senior employees in this workplace?<br>- As a leader, do you think about using these benefits?<br>- If so, how do you do this?<br>- Is it easy to do this? (what inhibits / promotes it?)<br>- Are these benefits further supported through courses, assignment of special responsibilities, etc.?<br>- Do you know whether the co-workers appreciate these benefits?<br>- What disadvantages does it have when employees get older in this workplace? (I.e. what disadvantages are associated with having employees who experience disabilities due to general aging, health problems or wear and tear?)<br>- How are these challenges handled?<br>- Is it something the employees themselves handle?<br>- In collaboration with their co-workers?

	- Is there something you can do as a manager or is there something the workplace does?
SENIOR POLICY	- Do you know if you have a senior policy in this workplace?<br>- If so - what does it contain?<br>- What opportunities does it give you as a leader?<br>- Do you use these opportunities in your department?<br>- If yes: Which parts do you use and how is it received?<br>- If no: Why not? (Ex: What are the challenges? How could this be solved?)
THE ROLL OF SENIOR EMPLOYEES AT THIS WORKPLACE	- Is this workplace generally interested in retaining senior employees?<br>- Why / why not?<br>- What status do employees over the age of 55 have in this workplace?<br>- Is this workplace generally interested in hiring senior employees?<br>- Why / why not?
FINAL COMMENTS	- In conclusion, is there anything you as a manager think is relevant in relation to this project, which we have not yet touched on?<br>- Is there anything else you would like to add before we end?

INTERVIEW GUIDE – FOCUS GROUP INTERVIEWS

PRESENTATION	- How old are you?<br>- What job function do you have in the workplace?<br>- How many years have you been in this workplace?<br>- Do you have the opportunity to organize your work / work tasks yourself?<br>(Instruction: You can draw on your own experiences, talk about it in general, or talk on the basis of knowledge that you have from others in the workplace)
CHALLENGES OF GETTING OLDER	- What are the challenges of getting older in this workplace? (physical, cognitive and emotional)<br>- When you experience these challenges in your work?<br>- What do you do then?<br>- How do you handle it in your daily life at work?<br>- Is there anything your colleagues, managers, workplace are doing/could do to deal with this?<br>- In a job like this, you often have a lot on your plaid (e.g. time pressure, new technologies etc.) - How do you handle this? -And is it a challenge when you get older?
SOK ELEMENTS (PROMOTING/ INHIBITING)	- Do you have the opportunity to get / use physical aids that can help you in your work?<br>- Is it possible to attend courses? /continuing education?<br>- Is it possible to spend more time on some work tasks?<br>- Is it possible to use a physiotherapist / training (can you train at work?)<br>- Is it possible to do other things that can help you in your work?
CHANGES IN WORK TASKE	- Do you believe that it is easier / harder to get older in your ward/work unit than other wards in the hospital/dairy? (why? / why not?)<br>- What are the positive things about getting older in this workplace?

- How do you use these positive things in your daily work?
- Are these positive things in demand by colleagues / manager / workplace?
-
- Are there tasks that you performed in the past that you do not perform today?
- If so, why/why not? (why did this change, on whose initiative did it happened, and how the new tasks work)
- Are there work tasks that you handle today that you have not had before?
- What is the background for this change in the work tasks?
- (Ask why this changed, on whose initiative it happened, and how the new tasks work)

- Are there work tasks you performed before that you no longer perform?
- Examples?
- What is the reason for this?
- Have you yourself decided no longer to handle these tasks?
- We asked for work assignments, but are there other functions (mentoring schemes, etc.) that you perform/ no longer preform?
- Has it been easy / difficult to change?

SENIOR POLICY

- Do you have a senior policy in this workplace?
- If so, do you know what this policy is about?
- Do you make use of the opportunities the senior policy provides?
- If so, which ones?
- If not, why not?
- Do you think they want employees to stay in this workplace for a long time?
- How do you experience that your colleagues, your immediate manager, your workplace generally perceive senior employees?

	- How is this expressed?
RETIREMENT	- Have you thought about when you would like to retire?<br>- What is your motivation for retiring/staying at work?<br>- What could make you stay longer in this workplace?<br>- What would it take you to both *be able* and *wanting to* stay in this job?<br>- Is it common to stay in this workplace for a long time?<br>- How old are people typically, when they retire from this job?<br>- What do you typically think is the reason people stop at this workplace?<br>- When employees stop at this job, do they typically retire or do they get a new job?
POSIBLE SOK IMPROVMENTS	- Is there anything you think you could do so that you as a senior employee can / want to stay in this workplace? (at the IGLO Levels/ in regards to the different types of SOC strategies?)<br>- Examples?<br>- Are these things used?<br>- How are they used?<br>- Why / why not?<br>- Is it possible to get courses, continuing education, etc.?<br>- Is it possible to make physical aids available?
FINAL COMMENTS	- In conclusion, is there anything you think is relevant in relation to this project of becoming older at work, which we have not yet touched on?<br>- Is there anything else you would like to add before we end?
